# Presenting signs of retinoblastoma at a tertiary level teaching hospital in Ethiopia

**DOI:** 10.11604/pamj.2017.28.66.11199

**Published:** 2017-09-22

**Authors:** Jemal Zeberga Shifa, Alemayehu Mekonnen Gezmu

**Affiliations:** 1 Department of Surgery, Faculty of Medicine, University of Botswana, Gaborone, Botswana; 2 Department of Paediatrics and Adolescent Health, Faculty of Medicine, University of Botswana, Gaborone, Botswana

**Keywords:** Retinoblastoma, leucocorea, proptosis

## Abstract

**Introduction:**

Retinoblastoma is a primary malignant intraocular neoplasm that arise from immature retinoblasts with in developing retina. The commonest presenting sign in developing country is proptosis which is the late presenting sign. We report presenting signs of retinoblastoma in Ethiopian children seen at a tertiary level teaching hospitals in Ethiopia.

**Methods:**

Prospective case series study was done on children who presented with retinoblastoma between May 1, 2005 and September 1, 2006. This study was done as part of requirement for partial fulfilment of certificate of specialty study in ophthalmology during the year 2005 to 2006. SPSS 11 statistical package was used to analyse the data.

**Results:**

Among 41 patients seen during the study period, 24 (58.5%) were males and 17(41%) were females. Unilateral retinoblastoma was found in 32 (78%) patients and bilateral cases were found in 9(22%). Mean age of onset for right eye was 27.5 months and left eye 33.7 months. The mean ages of presentation at time of diagnosis for right and left eye were 34.4 and 40.2 months, respectively .In bilateral retinoblastoma mean age of presentation was 33.3 months. The commonest presenting sign was proptosis 22(53.7%) followed by leucocorea nine (22%),ocular inflammation four (9.0 %), strabismus three (7.3%), glaucoma one (2.4%), loss of vision one (2.4%)and hyphemaone (2.4%).

**Conclusion:**

The commonest presenting signs of retinoblastoma in our set up were Proptosis followed by leucocorea. This is due to late presentation of patient and late referral by medical professionals. Health education to the public and health professionals will help early detection of retinoblastoma.

## Introduction

Retinoblastoma is a primary malignant intraocular neoplasm that arise from immature retinoblasts with in developing retina .It is the most common primary intraocular malignancy of childhood in all racial group [1–3]. Children with retinoblastoma frequently present with leucocorea [4, 5]. Prompt referral to ophthalmology and other paediatric specialists is necessary to optimize visual outcome and survival [5].

Retinoblastoma occurs in approximately 1 in 15,000 to 1 in 16,600 live births in the United States and Northern Europe. Retinoblastoma accounts for 11 percent of cancer in the first year of life. Between 2005 and 2009, the annual incidence of retinoblastoma in the United States among children younger than 15 years of age was 4.1 per million [6]. The median age at diagnosis is 18 months; an average of 12 months for children with bilateral disease and 24 months for children with unilateral disease [4]. Approximately 95 percent of children with retinoblastoma present before the age of five [3]. Nonetheless, cases of newly diagnosed retinoblastoma have been reported in children as old as 18 years [7–10] and rarely, even in adults [11]. The incidence is similar in boys and girls and among blacks and whites. A study done at university of Kinshasa showed that the most common presenting sign was leucocorea in 49 % of cases followed by proptosis in 28 % of cases , and bilaterally was observed in 21% of cases [12]. A similar study done at Chiang Mai university of Thailand showed that the most frequent presenting sign was leucocorea followed by proptosis , and bilaterality was observed in 37.7% of cases [13].

A study done in Addis Ababa university , medical faculty , Department of Ophthalmology about the pattern of eye lesion in children showed that the frequent intraocular as well orbital tumour was Retinoblastoma [14]. However, there was no research done in Ethiopia about the presenting sign of retinoblastoma.

During the last 10 years, there was significant changes in the treatment plan and approach for patients with intraocular retinoblastoma [15, 16]. This is due to an increasing number of patients with early detection of small tumours including the peripheral ones , more knowledge in the adverse effects of some treatments, such as external beam radiotherapy and advance in the use of systemic chemotherapy for child cancers [17].

In study done Argentina BuenosAires about late diagnosis of retinoblastoma in developing country, pediatricians are the first health professionals to evaluate most children with retinoblastoma. However, the diagnosis is not readily established. There is also a delay in consultation by parents, which is significantly longer in cases with advanced extra ocular disease [18, 19].

Retinoblastoma can be familial or sporadic. Germline mutations in the retinoblastoma (RB1) gene are present in approximately 40 percent of cases, predominantly in bilateral disease. Children with non-germline retinoblastoma incur new somatic mutations in one retinal cell from which the tumor arises. Less than 10 percent of retinoblastoma patients have a positive family history for the disease, suggesting that the majority of cases arise from somatic mutations and de novo germline mutations [20].

## Methods

A prospective case series study was done between May 1, 2005 and September 1, 2006 in Menilik II tertiary eye hospital, Addis Ababa, Ethiopia. This study was done as part of requirement for partial fulfilment of certificate of specialty study in ophthalmology during the year 2005 to 2006. The diagnosis of retinoblastoma was made based on history, clinical examination radiological and pathological study.

The history included age of patient, sex of patient, time of onset, sign and symptoms, family history of similar illness and any history of child death. Complete ocular examination and general examination were done by Ophthalmologist and paediatrician. Ultrasound examination of the fundus was made.

Pathological report of those patient who underwent surgery was recorded. A prepared format was used to record bio profile and findings for each patient. All children with diagnosis of retinoblastoma from May 1, 2005- September 1, 2006 were included in the study. Exclusion criteria were patients whose parents refused consent and pathology result which was other than retinoblastoma. The data was entered and analysed using SPSS version 11 software. Ethical clearance was obtained from research and publication committee of the department of ophthalmology at Menilik II hospital.

## Results

Among 41 patients who were seen during the study period, 24(58.5%) were males and 17(41%) were females. Unilateral retinoblastoma were found in 32 (78%), patients. Bilateral cases were found in nine (22%). All cases were sporadic, there was no family history of retinoblastoma noted. Mean of age onset for right eye was 27.5months and left eye was 33.7 months. For bilateral cases mean age of onset was 29.3months. The mean age of presentation of at time of diagnosis for right and left eye were 34.4 and 40.2 month respectively. In bilateral retinoblastoma mean age of presentation were 33.3 months.

The commonest presenting sign was proptosis 22(53.7%) followed by leucocorea nine (22%), ocular inflammation four (9. %), strabismus three (7.3%), Glaucoma one (2.4%), loss of vision one (2.4%), and hyphema one (2.4%).The diagnosis of retinoblastoma was made based on history, clinical examination, ultrasound examination and pathological examination were done in 31(75.6%) of patients.

The treatment given was as follows; 18(43.9%) of patients had exentration and chemotherapy, for 13(31.7%) of patients enouclation was done followed by chemotherapy and one case was treated outside Ethiopia with photocoagulation and external beam radiation. Three patients had distant metastasis and were sent to oncologist for chemotherapy. In Six patients family did not agree to have the operation so they were sent to oncologists.

## Discussion

The mean age of diagnosis for retinoblastoma in unilateral and bilateral cases in developed countries is 24 and 18 months respectively. In our study it was found to be higher with 27.5-33.7 months in unilateral and 29.3 months bilateral cases. This might be related to delay in presentation and /or delay in referral .The study which was done in Argentina Buenos Aires showed that there is a delay in diagnosis of retinoblastoma by paediatrician and delay from the side of the parents [19]. The other possible reason for the delay in the presentation is lack of awareness among health professionals, which leads to delay in timely referral to ophthalmologist. Leucocorea was found to be the commonest presenting sign in developed countries. But in our study the commonest presenting sign was proptosis followed byleucocorea. In this study bilateral retinoblastoma was detected in 22%, which is similar to other study done in Africa [12].

Untreated retinoblastoma is a deadly disease. The tumors grow to fill the eye and destroy the globe [21]. Metastatic spread may begin within 4 months of diagnosis, and death can occur within a year following metastasis. In the United States, with treatment, the survival rate for retinoblastoma is greater than 95 percent [22].

The most common routes of metastatic spread are direct infiltration via the optic nerve to the central nervous system, or spread via the choroid into the sclera and into the orbit [23]. Additional routes of spread include dispersion of the tumor cells through the subarachnoid space to the contralateral optic nerve or through the cerebrospinal fluid to the central nervous system; haematogenous dissemination to the lung, bone, liver, or brain; and lymphatic dissemination if the tumor spreads anteriorly into the conjunctivae, eyelids, or extra ocular tissue. While cure rates for orbital recurrences remain high, the mortality rate for extra-orbital metastatic disease is greater than 50 percent [24, 25].

The choice of treatment depends upon visual prognosis, tumor size and location, presence or absence of vitreous or sub retinal seeds and patient age. Standard therapeutic options include enouclation, chemotherapy, external beam radiation therapy, radioactive plaques (I-125 brachytherapy), cryotherapy, and laser photoablation [17, 26]. In 18(43.8%) of our patients, due to the late appearance to ophthalmologist the tumor had involved the orbit and exentration were done and they were sent to oncologist for chemotherapy. Three of our patients had distant metastasis and were sent to oncologist for chemotherapy.

### Limitations of the study

The following limitations are identified in the study: A) This is a hospital base study and the sample size is very small to make generalised conclusion; B) This study was done in 2005-2006. The result may not reflect what is happening currently.

## Conclusion

From our study most of our patient came with proptosis which showed that the public and the health professional were not aware of early signs and symptoms. Creating awareness among the public and the professional is needed to salvage the life and vision of patients with retinoblastoma.

### What is known about this topic

- The commonest presenting sign of retinoblastoma in developed world is leucocorea;
- Late presentation of retinoblastoma is not uncommon in developing world.

### What this study adds

- Early detection of retinoblastoma has paramount importance in the outcome of retinoblastoma patient management;
- Late presentation and detection has increased morbidity and mortality in patients with retinoblastoma.

## Competing interests

The authors declare no competing interests.
